# Liquid Native MALDI Mass Spectrometry for the Detection of Protein-Protein Complexes

**DOI:** 10.1007/s13361-018-2015-x

**Published:** 2018-07-31

**Authors:** Martine Beaufour, David Ginguené, Rémy Le Meur, Bertrand Castaing, Martine Cadene

**Affiliations:** 1 Centre de Biophysique Moléculaire, UPR4301, CNRS, affiliated to Université d’Orléans, Rue Charles Sadron, 45071 Orléans Cedex 2, France; 2 Present Address: Department of Biochemistry, Vanderbilt University School of Medicine, Nashville, TN USA; 3 Center for Structural Biology, Vanderbilt University, Nashville, TN USA

**Keywords:** Native mass spectrometry, Native MALDI, Nondenaturing conditions, Matrix-assisted laser desorption ionization, Liquid matrix, Ionic matrix, Noncovalent complexes, Protein-protein interactions, Protein-ligand interactions, HU protein, Streptavidin, Biotin

## Abstract

Native mass spectrometry (MS) encompasses methods to keep noncovalent interactions of biomolecular complexes intact in the gas phase throughout the instrument and to measure the mass-to-charge ratios of supramolecular complexes directly in the mass spectrometer. Electrospray ionization (ESI) in nondenaturing conditions is now an established method to characterize noncovalent systems. Matrix-assisted laser desorption/ionization (MALDI), on the other hand, consumes low quantities of samples and largely tolerates contaminants, making it a *priori* attractive for native MS. However, so-called native MALDI approaches have so far been based on solid deposits, where the rapid transition of the sample through a solid state can engender the loss of native conformations. Here we present a new method for native MS based on liquid deposits and MALDI ionization, unambiguously detecting intact noncovalent protein complexes by direct desorption from a liquid spot for the first time. To control for aggregation, we worked with HUαβ, a heterodimer that does not spontaneously rearrange into homodimers in solution. Screening through numerous matrix solutions to observe first the monomeric protein, then the dimer complex, we settled on a nondenaturing binary matrix solution composed of acidic and basic organic matrices in glycerol, which is stable *in vacuo*. The role of temporal and spatial laser irradiation patterns was found to be critical. Both a protein-protein and a protein-ligand complex could be observed free of aggregation. To minimize gas-phase dissociation, source parameters were optimized to achieve a conservation of complexes above 50% for both systems.

**Electronic supplementary material:**

The online version of this article (10.1007/s13361-018-2015-x) contains supplementary material, which is available to authorized users.

**Graphical Abstract:**
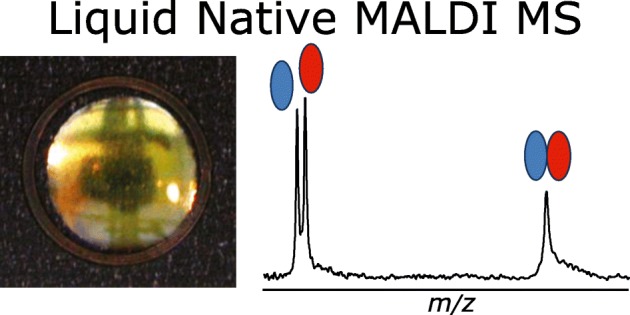
ᅟ

## Introduction

Native mass spectrometry (MS) methods consist in directly analyzing biological molecular assemblies in an instrument. By definition, they imply the conservation of the noncovalent interactions initially present in solution all the way to the detection stage. Approaches for the direct detection of noncovalent protein complexes by MS have been developed for over two decades, becoming a useful tool in structural biology and structure-activity relationship studies. Compared to other structural approaches, MS does not require extensive sample preparation, consumes much smaller quantities of sample, and yields results very rapidly, making it ideal for preliminary structure information and quaternary structure determination [1–3].

So far, the go-to ionization method for native MS has been electrospray (ESI). Although evidence of noncovalent complexes was shown for both ESI-MS [4–6] and MALDI (matrix-assisted laser desorption/ionization) MS around 1990 [7, 8], the rapid expansion in number and scope of native MS applications can be credited to ESI-MS methods [1, 2]. Yet, several arguments plead for MALDI development in native MS, as sample consumption is even lower than in ESI, and tolerance towards contaminants and buffers is greater. The use of additives, including nonvolatile compounds such as glycerol and diethanolamine, is possible, giving more leeway to tailor the matrix solution composition to a given system. MALDI’s tolerance to nonionic and zwitterion detergents for example, as demonstrated in MALDI in denaturing conditions, should facilitate the analysis of membrane protein complexes.

Our aim in developing such a native MALDI method is to be able to follow protein-protein or protein-ligand binding. Applications should eventually range from the fine characterization of protein-protein complexes to the monitoring of drug-target association to assess the specificity, selectivity and stoichiometry of binding, and finally, the elucidation of the mechanism of formation of enzyme-inhibitor or nonenzymatic complexes.

To directly make the method biologically relevant, we chose to develop the native MALDI method directly for whole proteins. For proof of concept, protein complexes in the category of a protein-protein complex and a protein-ligand complex should be observed. The main requirement is to be able to analyze noncovalent complexes in conditions that mimic biological solutions.

**Scheme 1:**
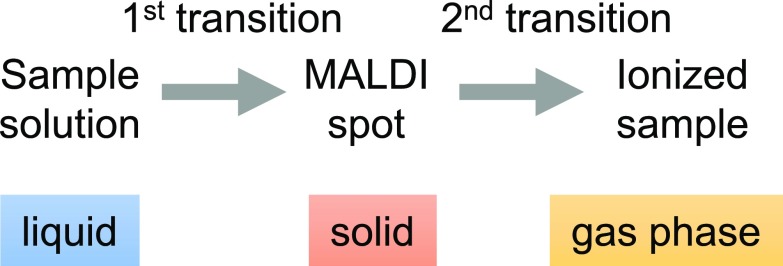
In solid MALDI, a sample goes through two phase transitions

So far, native MALDI MS methods developed for protein complexes were based on solid deposits. By design, in these methods, the sample has to transition through a solid state, followed by transfer to the gas phase during desorption (Scheme 1).

**Scheme 2:**
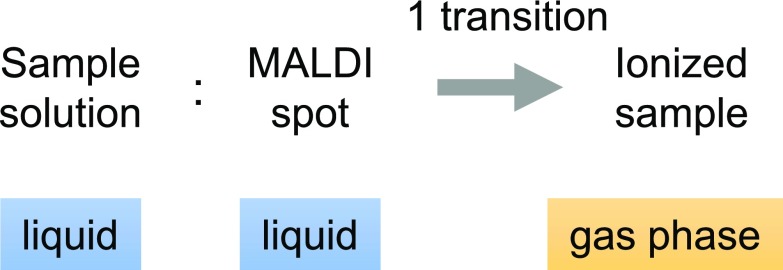
In liquid MALDI, a sample goes through a single phase transition

The rapid passage of complexes through a solid state raises questions as to the preservation of the native conformation of proteins and, thus, the biological relevance of the result. In this paper, we propose using liquid MALDI deposits in nondenaturing conditions to eschew the solid phase transition, leaving vaporization of the sample as the only transition (Scheme 2). Not only does this approach bring us closer to the well-established conditions of native ESI, it also mimics biological conditions more closely right up to the ionization stage, provided organic solvents are prevented from contact with the analyte complex.

Our strategy consisted in selecting the right matrix mixture by drawing from the corpus of existing liquid MALDI methods. The goal was to make a matrix solution that is homogeneous and stable over the course of analysis and that allowed us to see the protein complex. To increase the probability of observing an intact complex, we chose systems with very low equilibrium dissociation constants. Once obtained, we ensured that the complex signal is stable.

Next, in order to minimize the risk of aggregation, optimizations were driven with the aim to be sensitive enough to work at micromolar protein concentrations. The HUαβ native heterodimer protein was chosen because it does not readily rearrange into homodimers in solution [9], so that specific complexes initially formed in solution can be distinguished from gas-phase aggregates. To control for gas-phase dissociation, we set a goal to reach at least 50% conservation for both HUαβ and the streptavidin-biotin tetramer complex, which we achieved by focusing on instrument parameters optimization.

To date, this is the first report on the detection of specific noncovalent protein complexes originating from a liquid MALDI spot.

## Materials and Methods

### Materials

Recombinant HUαβ heterodimer from *Escherichia coli* was purified in-house as previously described [9]. Alkaline protease-treated streptavidin from *Streptomyces avidinii* was purchased from Promega (Madison, WI, USA). Myoglobin, ubiquitin, cytochrome c, and biotin were obtained from Sigma-Aldrich (Saint Quentin Fallavier, France). The matrices 4-hydroxy-α-cinnamic acid (HCCA), dihydroxybenzoic acid (DHB), and sinapinic acid (SA) were from Bruker Daltonics (Bremen, Germany). All other matrices, i.e., 9-aminoacridine (9AA), aniline (ANI), 2-nitrophenol (2NPHL), 6-aza-2-thiothymine (ATT), 3-aminoquinoline (3AQ), 4-aminoquinaldine (4AQA), and picolinic acid (PLA), were purchased from Sigma-Aldrich. Ammonium hydroxide was procured from Fluka (Steinheim, Germany), while diethanolamine (DEOA) and glycerol (Gly) were from Sigma. Ammonium acetate was from Merck (Darmstadt, Germany) and acetic acid from Thermo-Fisher Scientific (Illkirch, France). The organic solvents acetonitrile and methanol were purchased from Carlo Erba (Milan, Italy). All solvents and buffers were prepared using 18 MΩ water purified with Milli-Q reagent grade system from Millipore (Bedford, MA, USA), herein referred to as “ultrapure water.”

### Sample and Matrix Preparation for Native MALDI-TOF MS Analysis

**Figure 1:**
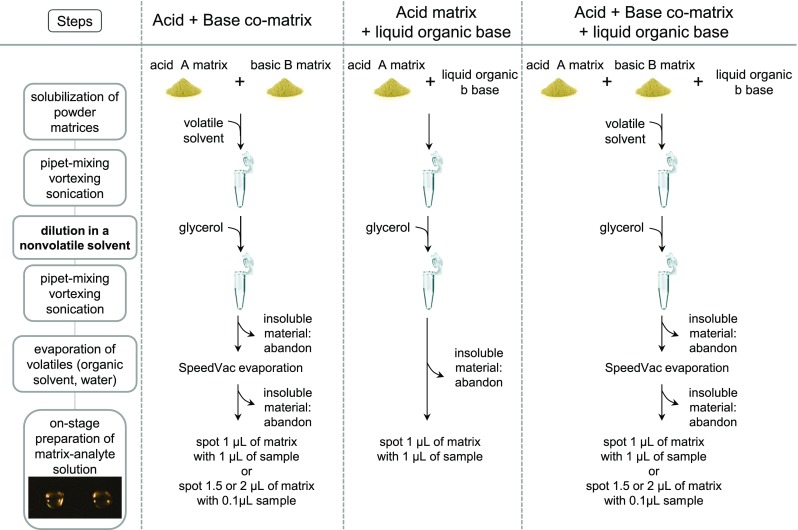
General procedure for the preparation of liquid matrices

Micromolar range HUαβ dimer solutions were prepared by dilution into glycerol from 1 mM stock solutions in 350 mM ammonium acetate, pH 5. The diluted samples were mixed 1:1 on-stage with liquid matrix solutions. Streptavidin tetramer solution was prepared at a concentration of 4 μM in 150 mM of ammonium acetate buffer, pH 6.9. The streptavidin-biotin complex was formed using a 4:1 ratio of biotin over streptavidin tetramer in 200 mM ammonium acetate, pH 7. Unless otherwise noted, the samples were diluted 50% with glycerol before 1:1 on-stage mixing with 1 μL of matrix solution. The general procedure we developed to prepare liquid matrices aimed at noncovalent complex analysis is presented in Fig. 1. The analyte-matrix samples were spotted onto a MTX stainless steel sample stage with final concentrations of protein complexes in the deposits in the 0.25–1 μM range. Calibrants were spotted using the same methods as samples.

### MALDI-TOF Mass Spectrometry

**Scheme 3:**
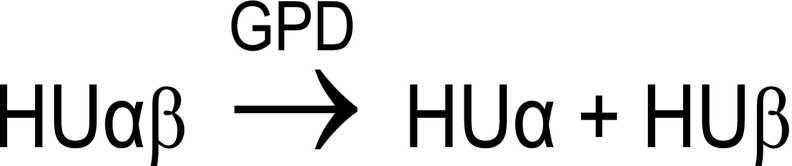
Gas phase dissociation in the case of HUαβ dimer

MALDI-TOF MS analyses were performed using an Ultraflex I MALDI-TOF/TOF mass spectrometer from Bruker Daltonics equipped with a 337-nm nitrogen laser and a gridless delayed extraction ion source. A deflection of matrix ions up to 2000 Da was applied to prevent detector saturation. Spectra were acquired with a 25-kV acceleration voltage in linear positive and/or negative ion mode by accumulation of 100–1400 laser shots. For HUαβ dimer, a 3000 to 41,000 *m*/*z* window was applied. For the streptavidin-biotin complex, spectra were acquired in the 9000 to 60,000 *m*/*z* range or in the 38,000 to 118,000 *m*/*z* range. The instrument was controlled using Bruker FlexControl software v3.3. Calibration was performed externally using myoglobin and ubiquitin for HU dimer, or a solution of apomyoglobin, cytochrome c, and 20–70 kDa Bruker calibrants for streptavidin-biotin complexes. MALDI-TOF-MS spectra were processed using FlexAnalysis software v3.3 (Bruker). To evaluate gas-phase dissociation (GPD) of oligomers occurring in the ion source, a percentage of remaining oligomers was calculated. Thus, HUαβ dimer can dissociate according to Scheme 3:

Equation (1) was used to approximate the percentage of conserved HUαβ heterodimer (%D) where *A* stands for peak area.

1 $$ \%\mathrm{D}=\frac{A_{\mathrm{HU}\alpha \beta}}{\frac{A_{\mathrm{HU}\alpha }+{A}_{\mathrm{HU}\beta }}{2}+{A}_{\mathrm{HU}\alpha \beta}}\times 100 $$

**Scheme 4:**
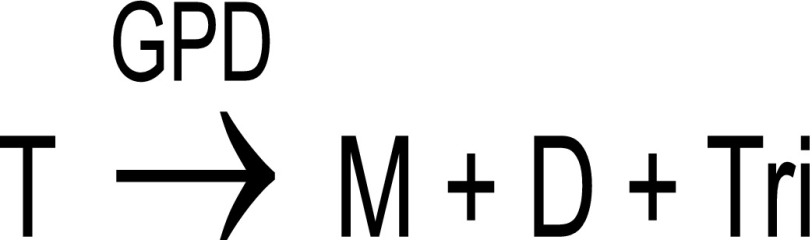
Gas phase dissociation in the case of streptavidin tetramer

Similarly, streptavidin can dissociate into lower-number oligomers and monomer (Scheme 4) where M, D, Tri, and T represent monomer, dimer, trimer, and tetramer, respectively.

The percentage of the conserved streptavidin tetramer (%T) was calculated as follows:

2 $$ \%\mathrm{T}=\frac{A_{\mathrm{T}}}{\frac{A_{\mathrm{M}}}{4}+\frac{A_{\mathrm{D}}}{2}+\frac{3\times {A}_{\mathrm{T}\mathrm{ri}}}{4}+{A}_{\mathrm{T}}}\times 100 $$

These calculations were applied to singly charged ions which were the dominant species in all spectra, while difference in ionization efficiencies between oligomers and monomers was neglected.

### ESI UHR-Q/TOF Mass Spectrometry

For native ESI-MS acquisitions, sample solutions of tetramer proteins were prepared at 5 μM in 150 mM ammonium acetate, pH 6.9, for streptavidin and the streptavidin-biotin complex. High-resolution ESI-MS was performed on a Bruker Maxis 4-GHz ESI-UHR-Q/TOF mass spectrometer piloted with micrOTOFcontrol v.3.2. Samples were introduced using direct infusion through a syringe at 1.3 μL/min with a nebulizer gas pressure of 0.7 bar and a drying nitrogen gas flow of 6 L/min. The spray needle voltage was − 4 kV with a − 500 V plate offset applied. The heated glass capillary temperature was set at 140 °C.

For HUαβ, the optimum isCID (in-source collision-induced dissociation) energy was 65 eV. The ion cooler transfer time and prepulse storage time were optimized at 115 and 33 μs, respectively. Spectra were acquired by summing scans for 40 s from *m*/*z* 300 to 3500. For the streptavidin-biotin complex, isCID energy was set at 10 eV, with transfer and prepulse storage times of 135 and 45 μs, respectively. Spectra were acquired by summing scans for 60 s from *m*/*z* 2500 to 4500. The instrument was calibrated externally with ESI-L Low Concentration Tuning Mix (Agilent Technologies). When applied, internal lock-mass calibration was based on hexakis(2,2-difluoroethoxy)phosphazene (Apollo Scientific, USA) at *m*/*z* 622 and hexakis(1H,1H,4H-hexafluorobutyloxy)phosphazine) at *m*/*z* 1222 (Agilent). Bruker Data Analysis v.4.0 was used for data treatment and deconvolution.

## Results

### Search for a Liquid Matrix to Go from a Protein Signal to the Observation of Noncovalent Protein-Protein Complexes

**Figure 2:**
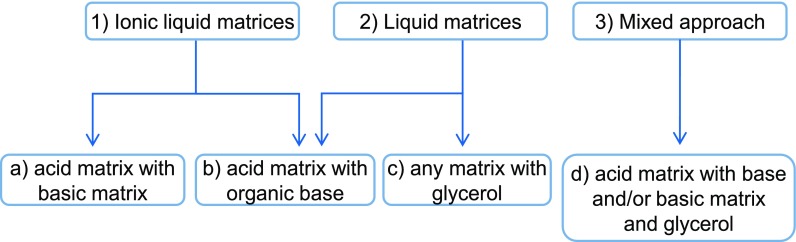
Strategies to obtain liquid matrices for use in native MALDI methods

We started our investigations by using the HUαβ protein. HU proteins are known as nonsequence-specific DNA-binding proteins and exist predominantly in *Escherichia coli* cells as an αβ heterodimer. The amino acid sequences of the α and β monomers as reported in Fig. S1 give a theoretical average mass of 9535 and 9226 Da, respectively, while the αβ heterodimer has an average mass of 18,761 Da. A highly purified preparation of the heterodimer was used for method development [9]. Since our strategy involves using a matrix in liquid form, we first investigated current methods for making liquid matrices for MALDI instruments with a 337-nm laser (Fig. 2). The most common method consists in mixing a matrix bearing an acid function with an organic base (Fig. 2, box b) [10–12] or an acid matrix with a basic matrix (Fig. 2, box a) [13]. The components are solubilized using volatile organic solvents until a completely homogeneous liquid phase is obtained. The ubiquitous HCCA and DHB matrices are often used for this approach. Naturally, this method only produces denaturing liquid matrices due to the presence of organic solvents. However, since a acid matrix could potentially form a liquid ionic matrix with a base, we decided to attempt this path, by evaporating the organic solvent after solubilization (Fig. 2, box b).

For this purpose, we used aniline or dimethylaniline, two readily available aromatic bases with boiling points of 184 and 194 °C, respectively, in combination with HCCA or DHB. In our hands, direct solubilization of the matrix with the base does not give a homogeneous ionic liquid. Alternatively, the matrix powder was solubilized in methanol with the base, and the methanol solvent was evaporated by SpeedVac. This approach however fails to produce homogeneous liquids.

We thus decided to substitute part of the methanol for a nonvolatile solvent in order to maintain the acid matrix-organic base mixture in liquid form after methanol evaporation (Fig. 1, first column). Of the very few nonvolatile solvents which meet with the nondenaturing criterion for native MALDI analysis, we chose glycerol. While in solid MALDI deposits, it is considered a contaminant adverse to crystallization and ionization, this is not an issue in liquid MALDI. Glycerol is used as a matrix in IR-MALDI [14] and has previously been used in liquid matrices [13, 15, 16] (Fig. 2, box d). Moreover, several of its properties make it a good candidate for development. Its near neutral pH and its known gentleness towards proteins in particular, leading to its common use as a cryogenic agent, make it compatible with a wide range of biomolecular interactions. Its high viscosity also helps maintain the integrity of the spot droplet under vacuum. Although glycerol may have limited solubilization properties towards MALDI matrices, it helps with solubility simply by virtue of diluting matrix and base in the matrix mixture.

In the literature, HCCA/ANI matrices (Fig. 2, box b) have been observed as powders [17]. In a first attempt at solubilization, mixtures of HCCA/ANI/Gly in ratios ranging from 1:1:2 to 1:1:517 (*n*/*n*) were tested (data not shown). A pale yellow liquid matrix with fully solubilized HCCA is obtained at an optimum ratio of 1:1:171 (*n*/*n*), with HCCA at a final concentration of 15 mg/mL (Table 1). Under the instrument’s vacuum, however, the liquid spot flattens upon further evaporation, finally reaching an amorphous solid-like state (Table 2). We thus looked for alternative matrices. ATT diluted in ethanol has been used in solid MALDI for noncovalent complexes studies in negative ion mode [18]. ATT was dissolved in methanol and diluted in glycerol at different ratios (Fig. 2, box c, Table 1). From ratios of 1:1 to 1:10 (*w*/*w*), ATT/Gly produces only insoluble material. At a ratio of 1:12 and up to 1:18 (*w*/*w*), the matrix stays liquid after methanol evaporation. A spotted droplet also stays liquid under vacuum. Negative ion mode analysis of HU dimer at a final concentration of 1 μM yields monomer protein peaks against a highly noisy background (data not shown). These conditions do not favor the observation of noncovalent complexes.

**Table 1:** Solubilization Assays for the Production of Liquid Matrices That Stay Liquid Under Vacuum and Produce HU Monomers Signal in MALDI-TOF MS

Matrix		Volatile solvent	Liquid organic base b	Nonvolatile solvent S	Proportions				Type of ratio	State after evaporation			State of deposit		Liquid matrix color	Mode	Monomer signal
Acid A	Base B				A	B	b	S		Solid	Liquid	Mixed	Solid	Liquid			
HCCA	–	ACN/H_2_O	ANI	Gly	1	–	1	171	*n*/*n*	–	X	–	–	X	No color	POS	Nonresolved
–	ATT	MeOH	–	Gly	1	–	–	1 to 10	*n*/*n*	X	–	–	n.d.	n.d.	n.d.	n.d.	n.d.
–	ATT	MeOH	–	Gly	1	–	–	12 to 18	*w*/*w*	–	X	–	–	X	No color	NEG	Resolved
SA	9AA	MeOH	–	Gly	1	4	–	6	*w*/*w*	–	–	X	–	X	Red	NEG	Nonresolved
SA	9AA	MeOH	–	Gly	1	5	–	12	*w*/*w*	–	–	X	–	X	Red	NEG	Nonresolved
SA	9AA	MeOH	–	Gly	1	2.5	–	6 or 12	*w*/*w*	–	X	–	–	X	Red	NEG	Nonresolved
HCCA	4AQA	MeOH	–	Gly	1	7	–	8	*w*/*w*	–	X	–	–	(X)	Yellow	POS	Nonresolved
PLA	3AQ	MeOH	–	Gly	1	4	–	6	*w*/*w*	–	X	–	–	X	Yellow	POS	Resolved
HCCA	3AQ	MeOH	–	Gly	1	4	–	6	*w*/*w*	–	X	–	–	X	Yellow	POS NEG	Resolved
HCCA	3AQ	MeOH	–	Gly	1	2.5	–	6	*w*/*w*		X	–	–	X	Yellow	POS NEG	Resolved
HCCA	–	–	DEOA	Gly	1	–	2.5	19	*n*/*n*	–	X	–	–	X	Yellow	NEG	Resolved
SA	–	–	DEOA	Gly	1	–	2.5	19	*n*/*n*	–	X	–	–	X	Yellow	NEG	Resolved
SA	9AA	–	DEOA	Gly	1	0.6	2.5	19	*n*/*n*	–	X	–	–	X	Reddish orange	NEG	Resolved
HCCA	2NPHL	–	DEOA	Gly	1	1.1	3	9.5	*n*/*n*	–	X	–	–	X	Orange	POS	Resolved
HCCA	2NPHL	–	DEOA	Gly	1	1	3.3	19	*n*/*n*	–	X	–	–	X	Orange	POS	Resolved

Diethanolamine (DEOA), a nonvolatile weak base, was used at 9 M. (X): the matrix solution lost solubility over storage

*Gly* glycerol

We proceeded to study co-matrices made of an acid matrix mixed with a basic matrix in glycerol (Fig. 2, box d; Fig. 1, first column). A sinapinic acid (SA) and 9-aminoacridine (9AA) co-matrix in glycerol at ratios of 1:2.5:6, 1:2.5:12, 1:4:6, and 1:5:12 produces a reddish viscous liquid at a final concentration of 20 mg/mL of SA. The matrices at 1:4:6 and 1:5:12 ratios contain insoluble material, so that only supernatants can be spotted. However, for all ratios, adduct formation prevents the resolution of clear signals in negative mode. HCCA produces a liquid matrix in combination with 4-aminoquinaldine (4AQA) in a 1:7:8 HCCA/4AQA/Gly ratio which tends to precipitate over time. Next, a solution of picolinic acid (PLA), 3-aminoquinoline (3AQ), and glycerol at a ratio of 1:4:6 (*w*/*w*) was prepared (Table 1). This worked well for producing a liquid matrix. After mixing with HU dimer solution, the spot stayed liquid in the instrument. Desorption of HU dimer in positive ion mode yielded hints of intact dimer. However, desorption in these conditions requires a high laser fluence and produces a lot of adducts.

**Table 2:**
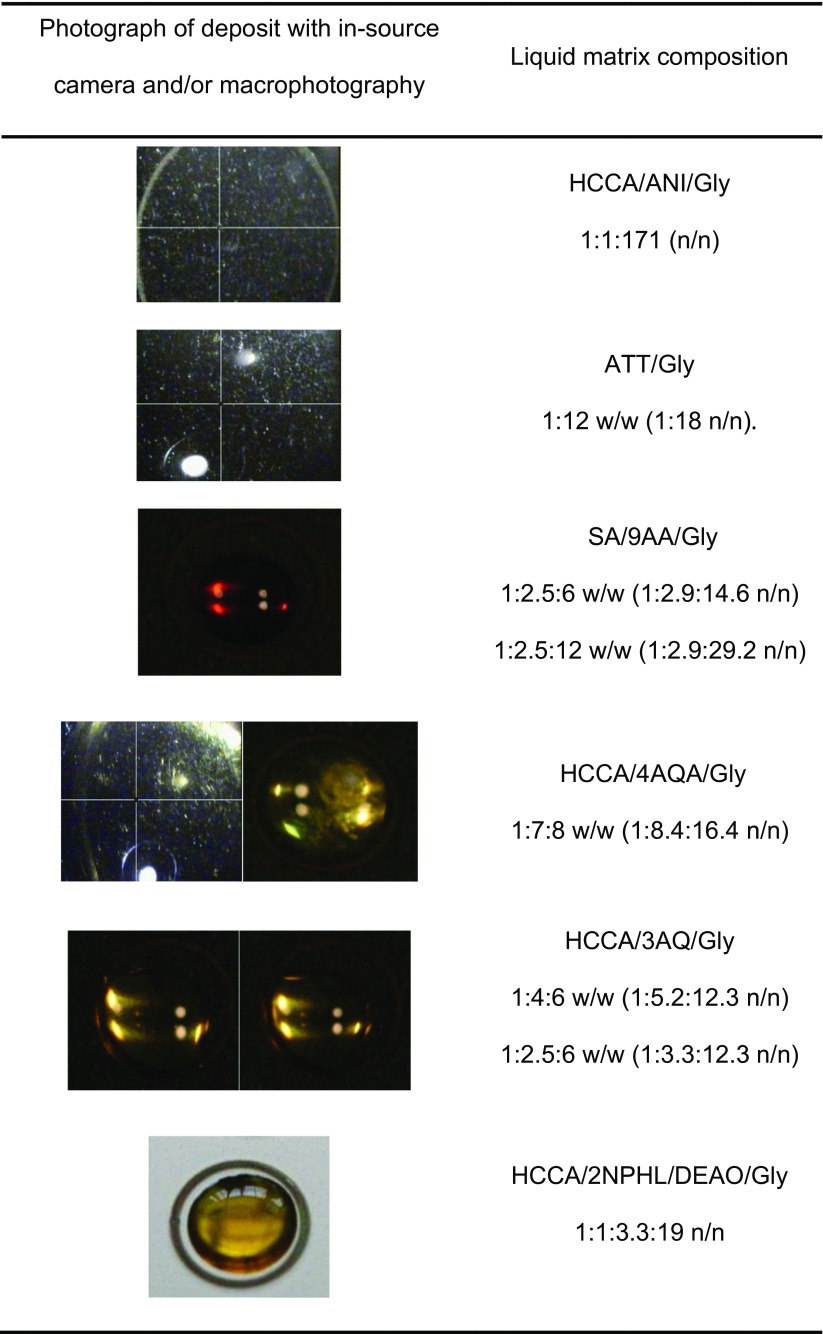
Liquid Spot Aspect Photographed with In-source Camera and Illumination and/or with Camera and Incandescent Lamp from Photo Reproduction Bench

In an era that predates the widespread use of solid two-layer and thin-layer deposition methods, Sze et al. developed ionic liquid matrices based on HCCA/3AQ/Gly with the hope of improving signal resolution and acquisition reproducibility compared to the dried droplet method [13]. At a ratio of 1:4:6 (*w*/*w*), a sharp rise in baseline toward low *m/z* values occurs. However, monomer HU peaks are clearly visible, and some dimer appears (not shown).

Finally, we devised a strategy that is essentially a combination of liquid ionic matrix (Fig. 2, box a) with the matrix-organic base approach (Fig. 2, box b) in addition to glycerol (box c), yielding a quaternary mixture (Fig. 2, box d). To keep any gained advantage in terms of evaporation, and to facilitate solubility during matrix preparation, a weak organic base with low volatility such as diethanolamine was considered. Various assays using HCCA, SA, 9AA, and 2-nitrophénol (2NPHL) were carried out (Table 1). In some cases, diethanolamine at 9 M is a good enough solvent in and of itself to make the use of methanol redundant. However, diethanolamine leads to serious adduction problems, so that it is preferable to maintain a large proportion of glycerol. The matrix solution SA/9AA/DEOA/Gly 1:0.6:2.5:19 (*n*/*n*) allows for well-resolved HU monomers observation but fails to allow for the detection of dimer. HCCA/2NPHL/DEOA/Gly produces resolved monomer peaks with few adducts but only at a ratio of 1:1:3.3:19 (*n*/*n*) and with a higher laser fluence than HCCA/3AQ/Gly (Fig. S2). It should be noted that this last approach seems to correspond to co-matrices in the liquid state, rather than true ionic liquid matrices, since, in our hands at least, it is not possible to obtain a liquid matrix after removal of methanol nor in the absence of DEOA.

**Figure 3:**
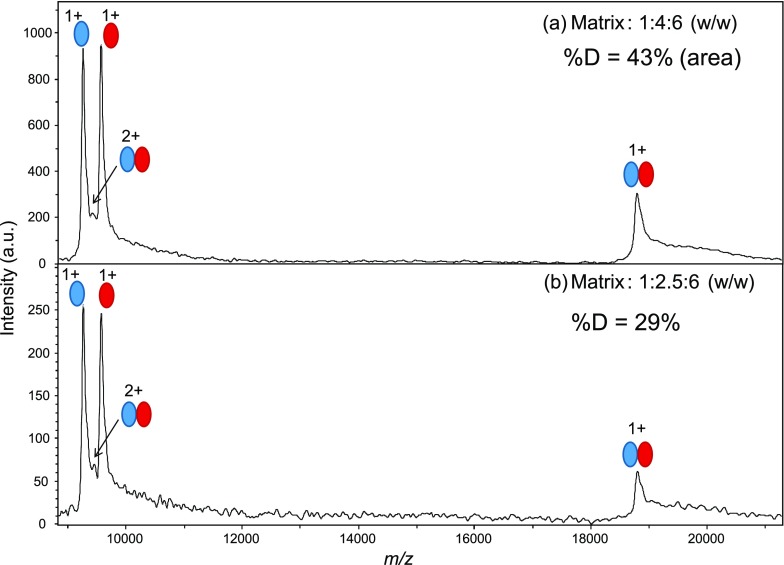
Liquid MALDI mass spectra of HUαβ in native conditions: effect of the composition of the HCCA/3AQ/Gly matrix. A solution of HUαβ at 0.5 μM in matrix was obtained by mixing sample solution 1:1 with matrix solution and deposited as a liquid spot. Analyses cumulated 200 laser shots. Red and blue ovals represent α and β monomers, respectively

A significant proportion of the HUαβ dimer is observed on mass spectra with the HCCA/3AQ/Gly liquid matrix (Fig. 3), in spite of gas-phase dissociation. To evaluate the conservation of dimer in the gas phase, we calculated the percentage of dimer %D, by picking areas of monomers and dimers as described in “Materials and Methods.” The unresolved doubly charged dimer peak was not taken into account in these calculations.

A distinctly lower %D (29 versus 43%) is obtained with a composition of 1:2.5:6 (*w*/*w*) versus 1:4:6. The latter gives the best combination of resolution, limited production of adducts, and stability under vacuum. The slightly acidic pH of this matrix (~ 5 measured with pH paper) is favorable for the conservation of the complex in the spot. It is sensitive enough to allow for careful monitoring of the signal for optimization. For the next steps in method development, it was thus chosen to optimize instrumental parameters.

### Optimization of Ion Source Parameters

**Figure 4:**
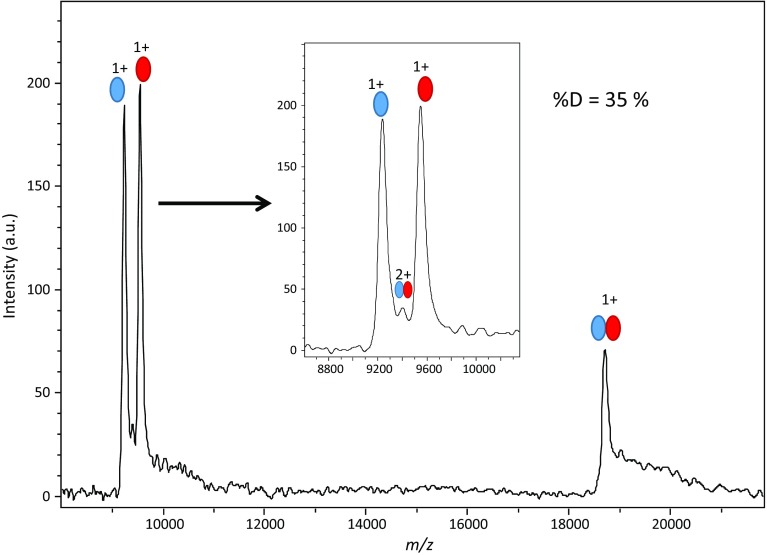
Analysis of HUαβ at *t*_ivr_ = 30 min. HUαβ was at a final concentration of 1 μM. Acquisition parameters were set at IS1 = 25 kV, IS2 = 23 kV, lens = 6 kV, laser intensity 39%, constantly moving laser shot pattern with a total of 200 shots

The sample/matrix liquid drop deposited on the sample stage was observed to undergo slow evaporation while in the ion source. The amount of time the sample stage spends in the source vacuum before acquisition is started could affect the signal. We thus examined the effect of this interval, defined as the in vacuo residence time (*t*_ivr_), on the %D. Spectra acquired at 20, 30, and 60 min resulted in %D values of 35, 35, and 36%, respectively, showing that the HUαβ dimer remains stable up to a *t*_ivr_ of 1 h. The spectrum obtained at a *t*_ivr_ of 30 min is shown in Fig. 4 for illustration. In contrast, the sample spot stability with the 1:2.5:6 (*w*/*w*) matrix composition shows a decrease of %D as a function of *t*_ivr_ (Fig. S3).

**Figure 5:**
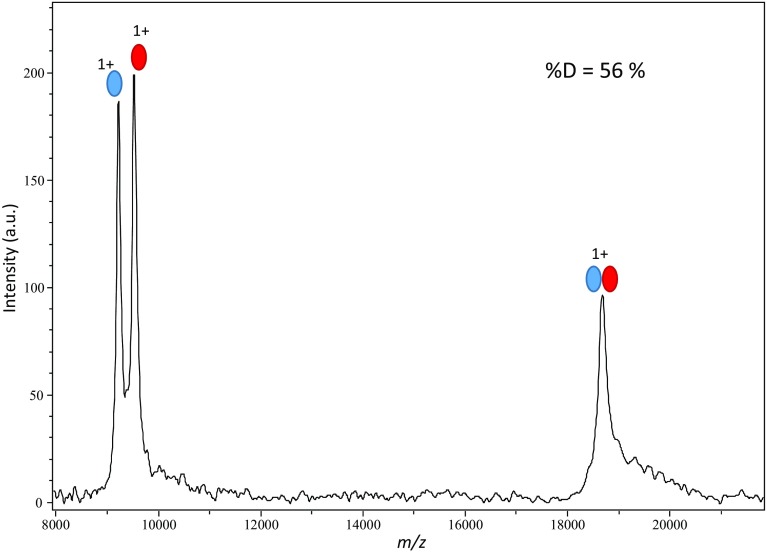
Analysis of HUαβ after optimization of source voltage parameters. HUαβ was at a final concentration of 1 μM. Final acquisition parameters were set at IS1 = 25 kV, IS2 = 23.45 kV, lens = 7 kV, laser intensity 36%, constantly moving laser shot pattern with a total of 200 shots

Ion source parameters have obvious effects on ion resolution and sensitivity. Some of them also influence ion energy, so that they could potentially affect the stability of noncovalent complexes. The delay was optimized for maximum resolution, as a compromise over the 9 to 20 kDa mass range (data not shown). A value of 400 ns allows for the simultaneous observation of singly charged HUαβ dimer and fairly resolved HU monomers. We proceeded to adjust the secondary ion source voltage IS2 and the lens voltage while keeping the primary voltage IS1 at a constant 25 kV. After several rounds of optimization of IS2 and lens voltage, the optimum parameters for the conservation of the HUαβ dimer were found at 23.45 and 7 kV for IS2 and lens voltages, respectively (Fig. 5). These new source parameters bring the %D from 35% (Fig. 4) to 56%. Prior to optimization, peak resolutions were 135, 133, and 163 for HUβ, HUα, and HUαβ, respectively (Fig. 4). In optimized conditions (Fig. 5), peak resolutions decrease slightly to 116, 124, and 110, respectively. Thus, for the HUαβ system, the stability of the complex is increased at the expense of the resolution. Since the doubly charged dimer is not taken into account in these calculations, the %D is underestimated.

In order to compare this method with an existing native MS approach based on solid MALDI-MS, HUαβ was analyzed using an aqueous solution of sinapinic acid matrix, as described by Song [19]. In our hands, at 1 μM of HUαβ dimer, no signal was observed, while at 10 μM, i.e., the upper limit of concentration that can be considered safe from gas-phase aggregation, only 10% dimer was conserved (Fig. S4).

### Spatial and Temporal Patterns of Laser Shots

Laser intensity can be adjusted not only for rising above ionization threshold and preventing detector saturation, but also for optimal dimer observation (Fig. S5). During development steps, we noticed that the heterodimer was observed by constantly moving the location of the laser on the drop. This prompted us to investigate the impact of other aspects of laser irradiation. The spatial/temporal pattern of laser shots was thus modified: instead of constantly moving the laser over the liquid spot, we used the “single-shot” method. Namely, the spectrum resulting from a single laser shot was acquired, then the laser was moved to a different location, a single-shot spectrum acquired again and summed with the first, and so on until a total of 200 shots were cumulated. Then, we devised a “ten-shot” method under the same principle, whereby we shot on the same location ten times before moving the laser to another location.

**Figure 6:**
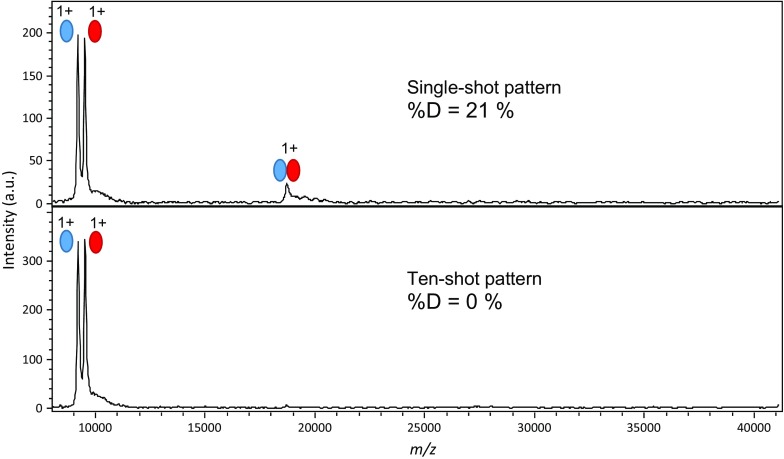
Comparison of single-shot versus ten-shot acquisition patterns: influence on HUαβ dimer stability. Acquisitions were performed in sets of one or ten laser shots before selecting another position on the liquid spot, with a total of 200 shots. Instrumental parameters were IS1 = 25 kV, IS2 = 23.75 kV, lens = 6 kV

As shown in Fig. 6, the stability of the dimer is strongly influenced by the spatial/temporal laser shot pattern. In fact, most of the dimer is lost when ten successive laser shots hit the same location on the spot. The marked increase in HUα and HUβ monomers confirms that HUαβ is essentially dissociated, with less than 5% dimer remaining. This experiment was done before final optimizations, so that the observed %D is quite low.

Even if the exact mechanism for dimer disruption is not well understood in this experiment, the ten-shot result serves as a negative control to validate the native MALDI MS result. Since there is no obvious way for gas-phase aggregation to be sensitive to the laser shot pattern, the difference between the single- and ten-shot results shows that the observed dimer is not the result of gas-phase aggregation. Moreover, the purified HUαβ heterodimer is an ideal system to discriminate noncovalent interactions from aggregation, as HUα_2_ and HUβ_2_ homodimers can only form in the gas phase. Neither HUα_2_ nor HUβ_2_ homodimer is formed (Figs. 3, 4, 5, and 6), confirming that the monomers are not prone to aggregation. Finally, Fig. 6 illustrates the absence of aggregates with masses in multiples of the dimer.

### Native MALDI MS of a Homotetramer—Small Molecule Complex

**Figure 7:**
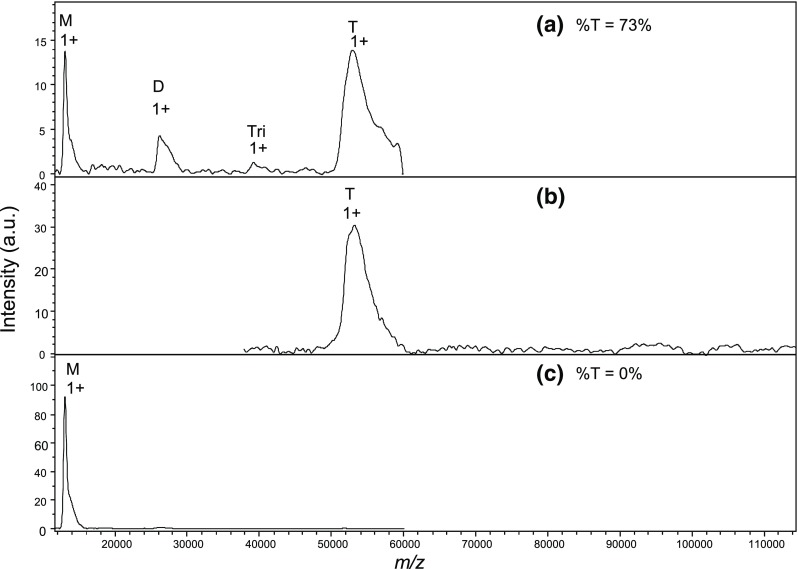
Comparison of single-shot versus ten-shot analysis of the streptravidin-biotin complex. The tetravidin-biotin complex was formed prior to mixing with HCCA/3AQ/Gly 1:4:6 matrix and analyzed at a final concentration of 0.5 μM streptavidin, 2 μM biotin. %T: Tetramer area percentage over all species. (**a**) Single-shot acquisition in the 12,000–60,000 *m*/*z* range. (**b**) Single-shot acquisition in the 38,000–114,000 *m*/*z* range. (**c**) Ten-shot acquisition in the 12,000–60,000 *m*/*z* range

To strengthen the evidence that noncovalent complexes can be observed in liquid MALDI, a second system was analyzed. The streptavidin-biotin complex was chosen for its very low equilibrium dissociation constant and the fact that it contains both protein-protein and protein-ligand interactions [20, 21]. A processed streptavidin was selected for its ability, according to the manufacturer, to exist as tetravidin (i.e., the tetrameric form of streptavidin) without biotin. This stock streptavidin is the product of nonspecific proteolysis at both the N- and C-terminal ends of the sequence by subtilisin, also known as alkaline protease, a proteinase with a preference for uncharged residues in P1. The manufacturer measured the mass of tetravidin at 53.2 kDa and calculated a corresponding mass of 13.3 kDa for free monomers. However, the exact sequence of the products is not provided. The amino acid sequence of the monomer from UniProt is shown in Fig. S6. We first analyzed this streptavidin in complex with biotin in conditions derived from optimizations with the HUαβ dimer. A peak of monomeric streptavidin is clearly observed with an average mass of 13,035.7 Da (Fig. 7a), far below the mass calculated by the manufacturer.

To determine which part of the sequence this mass corresponds to, we performed MALDI-TOF MS in denaturing conditions, where the monomer is not expected to retain any biotin. The monomeric streptavidin signal actually appears as four peaks with average masses ranging from 12,971.1 to 13,184.4 Da (Fig. S7). Based on the UniProt sequence, these masses are in perfect agreement with alternative cleavages at Ser 35, Ala 36, Ala 37, Ser 160, Ala 161, and Ala 162, consistent with the production of frayed ends by subtilisin cleavage of around two Ser-Ala-Ala sequences. The weighted average mass of the monomer of 13,059 Da will be used hereafter for the free monomer. The individual components of the peaks cannot be resolved in the native MALDI of Fig. 7 because of this protein sequence microheterogeneity so that (1) masses were determined as an average over the whole peak and (2) biotin stoichiometry calculations in native MS are rough approximations at best. As expected for this processed form of streptavidin, the observed monomer mass is consistent with the complete loss of biotin, while a peak at an average of 53,063 Da is observed around the expected mass for tetravidin (Fig. 7a). The width of the peak and the mass difference with the calculated tetravidin mass suggest that any bound biotin is only partially retained. Based on the monomer mass and an average biotin mass of 244.3 Da, additional peaks are consistent with the presence of smaller amounts of dimeric avidin (*M* = 26,263 Da) with about 0.6 bound biotins and with traces of trimeric avidin (~ 39.2 kDa).

The complex was also analyzed with a delay value of 700 ns and a lens voltage of 10 kV to focus on the mass range above the apparent tetrameric species (Fig. 7b), which confirmed the absence of aggregates. Finally, the dimeric, trimeric, and tetrameric peaks failed to appear with the ten-shot acquisition method (Fig. 7c), confirming the apparent dissociating nature of this laser shot pattern.

To compare behavior in liquid-state native MS, the stock streptavidin was also analyzed with and without 4 equivalents of biotin by ESI-UHR-QTOF MS in nondenaturing conditions. IsCID values were kept low to favor complex stability over complete desolvation, and data treatment adjusted to smooth over distributions linked to sequence heterogeneity.

**Figure 8:**
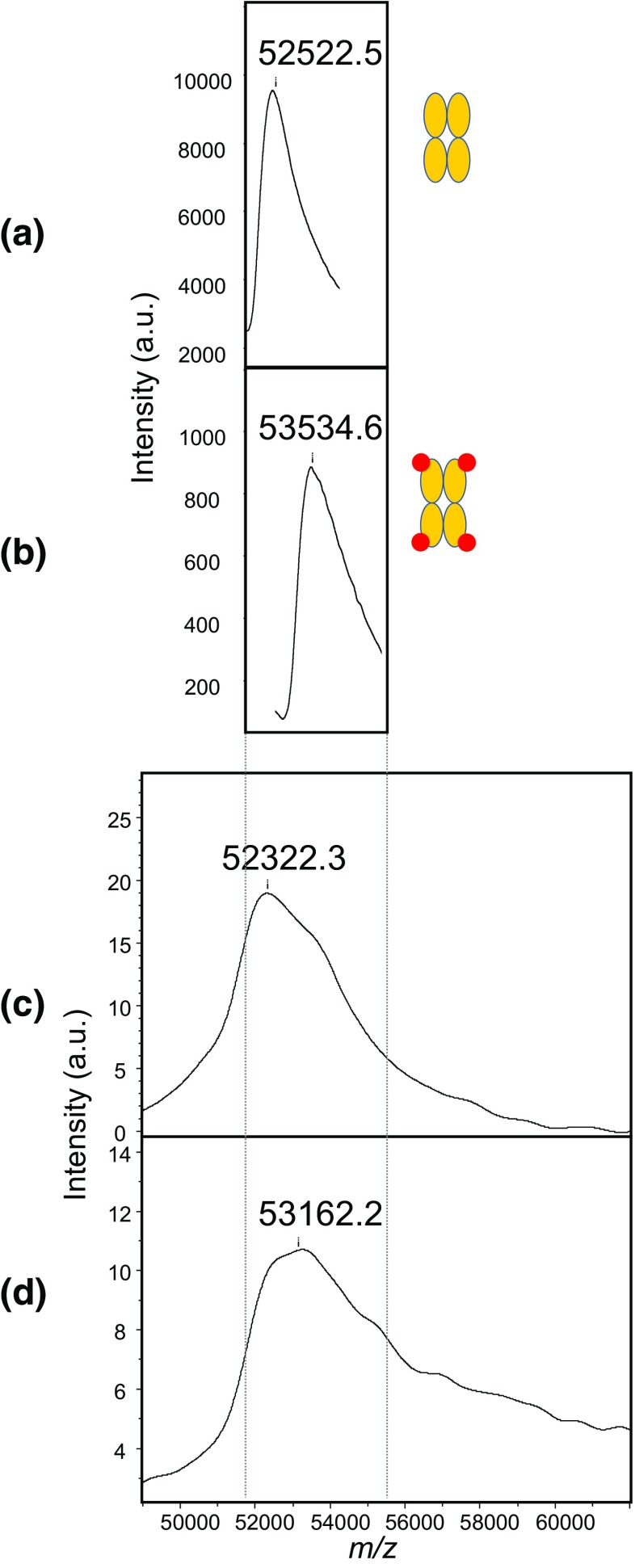
Tetravidin and tetravidin-biotin region in nondenaturing ESI-UHR-QTOF MS versus nondenaturing MALDI-TOF MS of streptavidin-biotin samples. Deconvoluted ESI-UHR-QTOF spectra of streptavidin at 2 μM in a solution of 150 mM ammonium acetate, pH 6.9, (**a**) in the absence of biotin and (**b**) as a preformed complex with 4 equivalents of biotin. (**c**) MALDI-TOF spectrum of streptavidin at a final concentration of 1 μM in 1:4:6 HCCA/3AQ/Gly matrix in the absence of biotin and (**d**) as a preformed complex of 0.5 μM streptavidin with 4 equivalents of biotin. Spectra were calibrated externally in the 44 to 66 kDa range using protein A and bovine serum albumin. Red circles represent biotin, while orange ovals represent streptavidin monomers

In the absence of biotin, the observed peak is wide (Fig. 8a), with a mass of 52,522 Da as determined by the sum peak algorithm, clearly above the theoretical tetramer mass calculated from the monomer mass (52,235 Da).

As expected, in the presence of biotin, the average tetravidin peak is shifted by 1012 Da (Fig. 8b), which is consistent with the binding of 4.1 molecules of biotin. The tetramer mass with and without the 4 bound biotins is a clear indication of partial solvation and/or adduct formation in native ESI. Liquid nondenaturing MALDI analyses were conducted on the same samples with narrow calibrant windowing around the tetravidin peak. The shift for deconvoluted peaks observed in ESI and for peaks obtained in liquid MALDI also indicates that in the absence of biotin, solvation/adduct formation of tetravidin occurs to a comparable extent (Fig. 8a, c). In native MALDI-TOF MS, however, the multimeric biotin-streptavidin complex appears as an even wider peak with a flattened top, averaging at a lower value than in native ESI (Fig. 8d), suggesting that some biotin dissociation occurs. A rough estimate of 3.4 apparent stoichiometry can be derived from the ~ 840-Da difference in sum peak masses. The greater dissociation of the biotin-tetravidin complex in MALDI may be due either to competition with at least one element of the matrix solution, or to absorption of extra energy brought about by laser illumination during ionization.

The lack of resolution precludes the clear characterization of the biotin-tetravidin species, so that the exact percentage of biotin-tetravidin dissociation remains unknown at this point. However, Fig. 7a shows that at 73%, the percentage of intact tetramer over the total of all forms is clearly above 50%.

## Discussion

MALDI methods for noncovalent complex analysis have been developed since the mid-1990s. As early as 1989, Karas and coworkers reported successfully detecting the tetramer of glucose isomerase using nicotinic acid diluted in water [7]. Despite these promising results, aggregation is also clearly present in these conditions, possibly owing to the biases inherent to the dried droplet method, i.e., locally high analyte concentration in crystals and/or the need for high laser fluence. In the following years, several groups worked on solid spot methods to analyze noncovalent complexes by MALDI, using a variety of matrices, matrix solution compositions, and deposition techniques (for reviews, see [22, 23]). The presence of organic solvents in the analyte solution and/or the matrix-analyte solution was shown to disrupt noncovalent protein complexes [24] or to favor aggregation of these complexes [25, 26]. The speed of matrix crystallization is incompatible with the crystallization of proteins so that complexes are likely embedded in amorphous form in matrix crystals.

In short, the fact that these methods were based on solid deposits goes some way to explain the skepticism they have evoked and therefore the lack of widespread acceptance in the field. In contrast, native ESI methods for the dissection of noncovalent complexes have now come of age [1, 2, 23]. To reboot method development in native MALDI analysis, we propose switching to liquid spot deposition. Although liquid MALDI methods have been developed from the very start of matrix-assisted ionization methods [27], they were conceived to improve shot-to-shot repeatability in direct analysis [11, 13] and in MALDI-MS imaging [12], and to develop quantitation methods [28, 29], rather than specifically for noncovalent protein complex analysis. Some liquid MALDI studies have shown smaller complexes such as peptide-metal adducts and an inclusion compound of phenylalanine within α-cyclodextrin [16]. In LILBID, a technique midways between MALDI and ESI, the laser desorption of noncovalent complexes from piezo-generated droplets of aqueous solution was demonstrated on a home-made LILBID-TOF instrument equipped with an IR laser [30, 31], albeit at fairly high analyte concentrations (~ 33 μM).

Here, we show for the first time that noncovalent protein-based complexes can be analyzed directly from the liquid phase by MALDI. The method is shown to work both for a protein-protein interaction and for a small molecule-protein oligomer complex. Picomole range quantities of noncovalent complexes are detected in the presence of HCCA/3AQ/Gly matrix, with concentrations in the low micromolar range. The mixing of acidic HCCA and basic 3AQ matrices gives a solution of slightly acidic pH, which should be compatible with a wide range of noncovalent assemblies. Interestingly, while the HCCA/3AQ ionic liquid is not miscible with water [32], it can be diluted in the more polar glycerol, giving a balanced mix of hydrophilicity and hydrophobicity which may promote the stability of complexes of molecules with differing polarities.

In the course of liquid native MALDI-MS development, we found that two main areas of instrument parameters were critical for success. The source voltages and the laser shot pattern can be adjusted to minimize the injection of potentially disruptive energy and to favor the conservation of the noncovalent complexes in the mass spectrometer. In ESI, the acceleration voltage, the interface pressure, and its heating temperature are optimized to reach a compromise between minimized gas-phase dissociation and improved desolvatation and transmission of the complexes. In the present study, the IS2 voltage (which contributes to acceleration) and the electrostatic focusing lens source voltage were tuned to better preserve intact complexes, leading to a sharp increase of %D for the HU heterodimeric protein.

Next, care is needed in choosing the temporal and spatial laser irradiation patterns to allow for the analysis of matrix-complex assemblies at the surface of the liquid spot. Soon after solid spot MALDI-MS was first used to study noncovalent complexes, Rosinke et al. discovered the first shot phenomenon [25]. It was later confirmed that signals related to noncovalent protein assemblies disappear [26] or decrease significantly for the shots following the first shot at a given position [33, 34]. Here, we show that the same effect can be obtained with a liquid spot. With the ten-shot pattern, the local thermal effect due to repeated absorption of the laser energy by the matrix-sample mixture seems to adversely affect noncovalent complexes. This could simply reflect a decrease in the affinity of the complex, based on the dependency of Gibbs free energy on temperature. Another possible interpretation is that an optimum spatial arrangement of matrix and protein complex molecules forms at the surface of the drop and that these clusters of “ideal geometry” are locally consumed upon desorption. With solid deposits, two independent studies using confocal laser scan microscopy showed that noncovalent complexes were localized at the crystal surface, i.e., with a specific arrangement in the vertical *z* axis of the droplet [34, 35]. The simplest explanation for this observation is that in solid deposition methods, localization of complexes at the surface happens upon droplet deposition on the sample stage, i.e., at the interface between the droplet and the atmosphere before the droplet dries. Moreover, intimate contact between analyte and matrix is necessary for desorption to work at the required level of sensitivity, so that noncovalent complexes at the surface are by definition surrounded by matrix molecules, and may form particular geometries with them in the *x* and *y* axes. As a direct consequence of both liquid and solid MALDI deposition methods going through the same liquid droplet deposition stage, we can then hypothesize that the ideal matrix-complex cluster arrangement mentioned above forms at the surface of the spot in solid MALDI as well.

The subsequent fate of these putative cluster arrangements however is the main difference between solid and liquid MALDI. With a solid deposit, any geometry of arrangement gets irreversibly frozen at the drying stage. For liquid MALDI, moving after every shot during single-shot accumulation could allow sufficient time for the ideal arrangement to regenerate over time. It also decreases the effective frequency of acquisition due to manually adding each spectrum to the acquisition, resulting in an overall cooler spot. Evidence of regeneration was found during acquisitions for this paper. In solid deposits, however, there is no possible diffusion to allow for regeneration. If so, this is another distinct advantage of liquid spot-based MALDI over solid spot-based native MALDI for the development of native MALDI MS.

Another potential advantage of this new method is that the viscous mixtures we use to apply native liquid MALDI are basically crowded media. There is growing interest at the interface between biology and physics in considering both the interior of a cell and the extracellular compartment as condensed matter and in exploring in which ways crowding of molecules can alter the behavior of biological systems [36]. Liquid MALDI characterization of complexes with matrices based on glycerol or viscous polymers could be useful tools for these studies.

Our initial goal of observing at least 50% of intact protein complex was reached for both systems, meaning that dissociation was kept below 50%. Gas-phase dissociation is dependent on the type of microscopic interactions involved in the protein-protein or protein-ligand system under study, as quantified by the *f*_sat_ parameter [37]. Consequently, a 100% complex conservation is probably achievable only for systems based purely on electrostatic interactions. HU, which forms dimers through a common hydrophobic core as shown both by crystallography for HUα_2_ [9] and by molecular modelization for HUβ_2_ [38], is probably not such a system.

However, there is room for improvement in fighting gas-phase dissociation. For example, the matrix composition could be tailored for each system according to the type of noncovalent interactions involved in the complex. This may bring the method to a degree of performance compatible with lower affinity protein-ligand systems. We could also consider inspecting the effect of the concentration of MS-compatible salts in the liquid spot, as was done in native ESI for HBsu, the *Bacillus subtilis* homolog of HU protein [39].

In conclusion, proof of concept has been established for both a protein-protein and an oligomeric protein-ligand complex. This work shows encouraging results for the future of liquid native MALDI MS. Such methods could have a large gamut of applications, over a wide range of masses with a suitable detector. The tolerance of MALDI to detergents means native MALDI MS could be applied to transmembrane and other hydrophobic proteins in a more straightforward way than is possible with ESI. Liquid native MALDI with a viscous matrix could have numerous applications in MALDI imaging including complex quantification, and allow multimodal detection with any number of compatible imaging techniques. Finally, comparing and contrasting MALDI and ESI methods for native MS could help us decipher mechanisms that are highly relevant to the conservation and detection of complexes in the gas phase, which in turn could help us further identify the critical points needed to keep improving these methods.

## Conclusion

This work shows encouraging results for the future of liquid native MALDI MS, establishing proof of concept for both a protein-protein and an oligomeric protein-ligand complex. A MALDI approach to nondenaturing MS bears obvious advantages. With MALDI-TOF instruments, a large gamut of masses is amenable to analysis. In addition, the small number of charges born by molecular complex ions makes data interpretation straightforward and gas-phase aggregation easier to identify. Moreover, MALDI’s inherent resistance to buffer components that are known to suppress MS signal in ESI, e.g., detergents, means that the analysis of membrane protein complexes may no longer be such a hard challenge.

## Electronic Supplementary Material

ESM 1 (DOCX 769 kb)
