# Supplementary material for: Liquid Native MALDI Mass Spectrometry for the Detection of Protein-Protein Complexes
Source: J Am Soc Mass Spectrom. 2018 Jul 31;29(10):1981–94. doi: 10.1007/s13361-018-2015-x (PMC6153977; doi:10.1007/s13361-018-2015-x)
Supplement: Supplementary file 1 — (DOCX 769 kb) [file 13361_2018_2015_MOESM1_ESM.docx]

**Supplementary materials to: Liquid native MALDI Mass Spectrometry for the detection of protein-protein complexes**, Beaufour *et al.*

**Figure S1. Amino acid sequences of HUα and HUβ *(Escherichia coli)***

**HUα**

10 20 30 40 50
MNKTQLIDVIAEKAELSKTQAKAALESTLAAITESLKEGDAVQLVGFGTF
 60 70 80 90
KVNHRAERTGRNPQTGKEIKIAAANVPAFVSGKALKDAVK

Average mass: 9535 Da

**HUβ**

10 20 30 40 50
MNKSQLIDKIAAGADISKAAAGRALDAIIASVTESLKEGDDVALVGFGTF
 60 70 80 90
AVKERAARTGRNPQTGKEITIAAAKVPSFRAGKALKDAVN

Average mass: 9226 Da

**Figure S2. Quaternary liquid matrix mixture for noncovalent dimer analysis.**

HUαβ protein 0.25 µM in HCCA/2NPHL/DEA/Gly 1:1:3.3:19 (n/n).

Laser intensity: 46%

0

10

20

30

40

1+

1+

1+

10000

12000

14000

16000

18000

20000

*m/z*

Intensity (a.u.)

**Figure S3. Effect of *in vacuo* residence time (t*_ivr_*) of the liquid spot in the source before acquisition.** HUαβ at 0.5 µM in matrix was deposited by mixing sample 1:1 with HCCA/3AQ/Gly 1:2.5:6 (w/w). Acquisition parameters were set at IS1 25 kV, IS2 23.35 kV, lens 6 kV, and laser intensity 46%.

0

20

40

60

80

100

0

50

100

150

200

250

300

0

200

400

600

800

1000

1200

10000

12000

14000

16000

18000

20000

*m/z*

1+

1+

2+

1+

1+

t

*ivr*

=15 min

%D = 39%

t

*ivr*

= 56 min

%D = 29%

t

*ivr*

= 267 min

%D = 25.6%

1+

1+

1+

1+

1+

2+

2+

(a)

(b)

(c)

Intensity (a.u.)

**Figure S4. Solid MALDI mass spectra of HUαβ using an aqueous dissolved matrix.** A solution of HUαβ at 10 µM in matrix was obtained by mixing sample solution 1:1 with matrix. Matrix solution: saturated sinapinic acid (SA) in 0.44 M ammonium citrate dibasic, adjusted to pH 5.1. Instrumental parameters: IS1 = 25 kV, IS2 = 23.45 kV, Lens = 7 kV (identical to those of Figure 5), laser intensity 46%, constantly moving laser shot pattern. * : SA adducts

**Figure S5. Effect of the laser intensity on the HUαβ noncovalent signal.**

HUαβ protein 0.5 µM in HCCA/3AQ/Gly matrix (1/4/6). Laser intensity at (a) 53 %, (b) 46 %.

1+

**Figure S6 : Amino acid sequence of streptavidin *(Streptomyces avidinii)***

Preferential subtilisin cleavage sites are highlighted with boldface and underlined. The protein form most frequently seen in structures corresponds to sequence 37-160.

10 20 30 40 50
MRKIVVAAIAVSLTTVSITASASADPSKDSKAQV**SAA**EAGITGTWYNQLG
 60 70 80 90 100
STFIVTAGADGALTGTYESAVGNAESRYVLTGRYDSAPATDGSGTALGWT
 110 120 130 140 150
VAWKNNYRNAHSATTWSGQYVGGAEARINTQWLLTSGTTEANAWKSTLVG
 160 170 180
HDTFTKVKP**SAA**SIDAAKKAGVNNGNPLDAVQQ

Average mass: 18834 Da

**Figure S7. Mass spectrum of streptavidin in denaturing conditions.**

Ultrathin layer analysis of streptavidin at 0.25 µM in HCCA/TWA (acetonitrile:water:trifluoroacetic acid 33.3:66.6:0.1) [SI1,SI2].

Intensity (a.u.)

References:

1. Gabant G., Cadene M. (2008) Mass spectrometry of full-length integral membrane proteins to define functionally relevant structural features. *Methods* **46**, 54-61.
2. Cadene M., Chait B.T (2000) A robust, detergent-friendly method for mass spectrometric analysis of integral membrane proteins. *Anal. Chem*. **72,** 5655-5658.

.
